# A domestication-associated gene, *CsLH*, encodes a phytochrome B protein that regulates hypocotyl elongation in cucumber

**DOI:** 10.1186/s43897-021-00005-w

**Published:** 2021-06-16

**Authors:** Bin Liu, Jinyang Weng, Dailu Guan, Yan Zhang, Qingliang Niu, Enrique López-Juez, Yunsong Lai, Jordi Garcia-Mas, Danfeng Huang

**Affiliations:** 1 School of Agriculture and Biology, Shanghai Jiao Tong University, Key Laboratory of Urban Agriculture (South), Ministry of Agriculture, Dongchuan Road, Shanghai, 200240 China; 2 Centre for Research in Agricultural Genomics (CRAG), CSIC-IRTA-UAB-UB, Campus Universitat Autònoma de Barcelona, 08193 Bellaterra, Spain; 3 College of Horticulture, Northwest A&F University, Yangling, 712100 Shaanxi P.R. China; 4 Department of Biological Sciences, Royal Holloway University of London, Egham, TW20 0EX UK; 5 Institute of Pomology & Olericulture, Sichuan Agricultural University, Chengdu, P.R. China

## Introduction

Plant height is an important agronomic trait; tall plants are prone to collapse (lodging) and are unsuitable for high-density planting (Li et al., 2020). During the Green Revolution, a multitude of genes acting as core or peripheral regulators of plant height were identified and used in breeding (Eshed and Lippman, 2019); however, most were reported in cereal crop plants (Eshed and Lippman, 2019) and few have been characterized in the Cucurbitaceae, which are economically important horticultural plants cultivated worldwide. Here, we describe *LONG HYPOCOTYL* (*CsLH*), encoding the photoreceptor phytochrome B (PHYB), which we show has been subjected to selection during cucumber (*Cucumis sativus* L.) domestication.

## Results

**Fig. 1:**
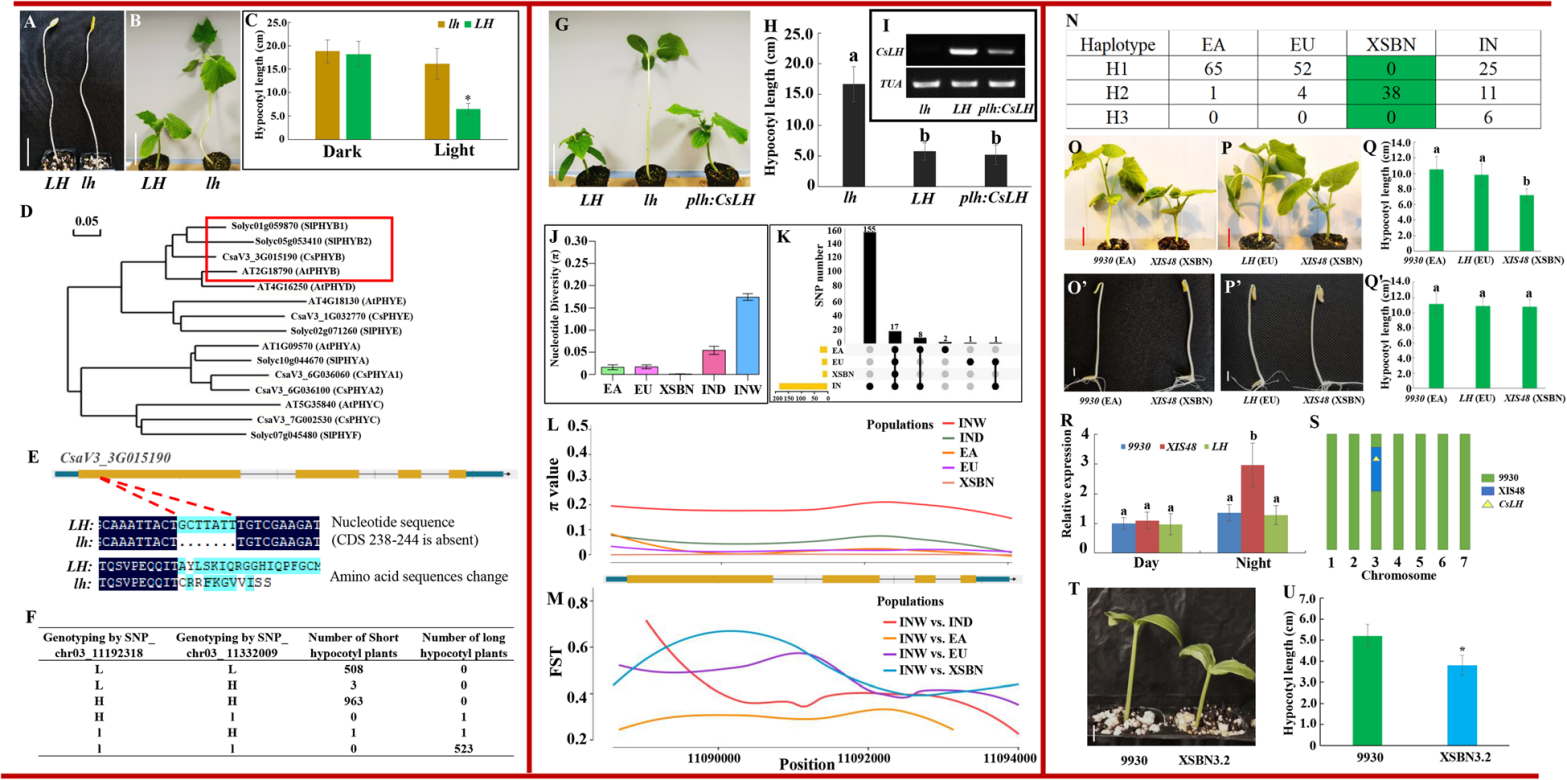
(**A**) Hypocotyls of 10-d-old wild type (*LH*) and *long hypocotyl* (*lh*) mutant seedlings under dark conditions. Bar, 5 cm. (**B**) Hypocotyls of 10-d-old *LH* and *lh* cucumberseedlings under white light conditions. Bar, 5 cm. (C) Hypocotyl length of the indicated genotypes in (**A**) and (**B**). Error bars indicate ± SD (*n* = 20). The asterisk indicates a significant difference (*P* < 0.05, *t* test). (**D**) Maximum likelihood tree of phytochrome protein sequences from *Arabidopsis thaliana* (At), tomato (*Solanum lycopersicum*; Sl), and cucumber (*Cucumis sativus*; Cs). The sequence data were deduced from amino acid sequences taken from the Arabidopsis Information Resource (https://www.arabidopsis.org/), Tomato Genome Database (https://solgenomics.net/), and Cucurbit Genome Database (http://cucurbitgenomics.org/). (**E**) The 7-bp deletion detected in the first exon of *CsaV3_3G015190* in the *lh* mutant; this deletion results in amino acid changes and a premature stop codon. (**F**) Genotyping of 2000 F_2_ individuals using two *CsLH* flanking markers: SNP_chr03_11192318 and SNP_chr03_11332009. L = homozygous SNP alleles from Chinese Long 9930 (reference genome); l = homozygous SNP alleles from *lh*; H = heterozygous SNP alleles. (**G**) *CsLH* rescued the long hypocotyl phenotype exhibited by the *lh* mutant at 10 days old. Bar, 5 cm. *plh: CsLH* = *CsLH* coding sequence driven by the *lh* promoter. (H) Hypocotyl lengths of the genotypes indicated in (**G**). Error bars indicate ± SD (*n* = 20). Different lowercase letters (**a, b**) indicate significantly different hypocotyl lengths in pairwise tests (*P* < 0.05, *Tukey’s* test). (**I**) Semi-quantitative RT- PCR analyses of *CsLH* expression in *lh*, *LH*, and *plh: CsLH*. The internal reference was the *α-TUBULIN (TUA)* gene. The primers for testing *CsLH* expression were designed based on the 7-bp deletion sequence; therefore, no band that could be amplified from the *lh* sequence. (**J**) Nucleotide diversity of the *CsLH* region in five cucumber groups, as measured using the π value. Error bars indicate ± SD. EA = East Asian, EU = Eurasian, IN = Indian cultivated and wild genotypes; IND = Indian Domestic, INW = Indian wild genotypes, XSBN = Xishuangbanna. (**K**) Matrix depicting unique and shared SNPs among the EA, EU, XSBN, and IN (including INW and IND) groups at the *CsLH* region. The yellow bars indicate total SNP number of each cucumber group. (**L**) Nucleotide diversity of five cucumber populations at individual nucleotide sites in *CsLH*. The *x* axis indicates the genome position in the Chinese Long 9930 v2 sequence, while the *y* axis indicates the π value of each cucumber group. (**M**) Pairwise difference of allele frequency (FST). The *x* axis indicates the genome position in Chinese Long 9930 v2, while the *y* axis indicates FST value of INW vs. IND, INW vs. EA, INW vs. EU, and INW vs. XSBN. (**N**) Number of haplotypes observed in each cucumber group. Green background highlight that XSBN only contained H2. (**O**) and (**P**) Representative EA (**O**) and EU (**P**) seedlings displayed longer hypocotyls than XSBN grown under white light conditions. Seedlings were 14 days old. Bar, 5 cm. (Q) Hypocotyl lengths of the indicated genotypes in (**O**) and (**P**). Error bars indicate ± SD (*n* = 20). Different lowercase letters indicate groups of significance difference in pairwise tests (*P* < 0.05). (**O′, P′**) Comparison of hypocotyl lengths among 7-d-old EA, EU, and XSBN seedlings grown under dark conditions. Bar, 1 cm. (**Q’**) Hypocotyl lengths of the indicated genotypes in (**O′**) and (**P′**). Error bars indicate ± SD (*n* = 20). Lowercase letters indicate no significant differences in the hypocotyl lengths of dark-grown EA, EU, and XSBN seedlings (*P* < 0.05). (**R**) Relative expression of *CsLH* under light and dark conditions in 9930, XIS48, and *LH*. Different lowercase letters indicate a significant difference (*P* < 0.05, *Tukey’s* test, three replications). (**S**) Model of the genome of the introgression line XSBN 3.2, as genotyped using SNP markers. Green and blue indicate background of parental line 9930 and XIS48, respectively. (**T**) Hypocotyl lengths of 5-d-old Chinese Long 9930 and XSBN3.2 seedlings grown under white light. Bar, 1 cm. (**U**) Hypocotyl lengths of indicated genotypes in (**T**). Error bars indicate ± SD (*n* = 20). Asterisk indicates a significant difference (*P* < 0.05)

The cucumber *long hypocotyl* (*lh*) mutant was previously identified as a light-dependent monogenic recessive mutant by Koornneef and van der Knaap (1983). To confirm this phenotype, we grew wild-type seedlings (*LH*) and *lh* under dark (Fig. 1a) and light (Fig. 1b) conditions. There was no difference in the hypocotyl lengths of *LH* and *lh* grown in the dark (Fig. 1a, c), while the *lh* mutant produced hypocotyls more than twice as long as those of *LH* when grown under white light (Fig. 1b, c),

López-Juez et al. (1992) reported that *lh* lacked a type-2 phytochrome-like polypeptide, a protein recognized by antibodies raised against a heterologous *PHYB* gene product from tobacco; however, the gene encoding this PHYB-like protein remains unknown. The recent publication of the cucumber genome (Li et al., 2019) has provided an opportunity to identify this gene. From the cucumber genome database, we identified five candidate *PHY* genes: *CsaV3_1G032770*, *CsaV3_3G015190*, *CsaV3_6G036060*, *CsaV3_6G036100*, and *CsaV3_7G002530*. We analyzed their phylogenetic relationships with *PHY* genes from *Arabidopsis thaliana* and tomato (*Solanum lycopersicum*) using their predicted protein sequences. *CsaV3_3G015190* was more closely related to the *Arabidopsis* and tomato PHYBs than were the other cucumber *PHY* genes (Fig. 1d). Next, we resequenced the genomes of *LH* and *lh* and found a 7-bp deletion in the 238–244 bp region of the *CsaV3_3G015190* coding sequence (CDS) in *lh*. This deletion disrupted the open reading frame (ORF), resulting in amino acid changes followed by a premature stop codon (Fig. 1e). Meanwhile, there was no difference in the other four *PHY* genes between *lh* and *LH*. *CsaV3_3G015190* is therefore the best candidate for the *CsLH* gene.

To test the role of *CsLH* at the population level, we crossed Chinese Long 9930 (reference genome, abbreviated as 9930) and the *lh* mutant and then self-fertilized the F_1_ generation to obtain an F_2_ population. Based on resequencing data, we developed two SNP markers between 9930 and *lh* that flanked the *CsLH* gene: SNP_ chr03_11192318 and SNP_chr03_11332009. For each F_2_ plant, if the SNP genotype corresponded to 9930, it was marked as L; if the SNP genotype was that of *lh*, it was marked as l; if the SNP genotype was hybrid (one allele of each kind), it was marked as H. In the genotyping of 2000 F_2_ individuals, we found 508, three, and 963 seedlings that showed the L^11192318^ L^11332009^, L^11192318^H^11332009^, and H^11192318^H^11332009^genotypes for the two SNPs, respectively, of which all exhibited the short hypocotyl (< 10 cm) of the 9930 parent. Meanwhile, the 523 plants that possessed *lh* alleles for both SNPs (l^11192318^l^11332009^) had the same long hypocotyl (> 10 cm) as the *lh* parent (Fig. 1f), indicating that *CsLH* confers the long hypocotyl phenotype. Next, we cloned the promoter of *lh,* followed by the CDS of *CsLH*, into the vector pEGAD and introduced it into the *lh* mutant using an *Agrobacterium tumefaciens*-mediated transformation. The long hypocotyl phenotype of the *lh* mutant was rescued in the three transgenic lines expressing *CsLH* (Fig. 1g–i).

Modern cucumbers have undergone a long domestication process, which has resulted in distinct groups of genotypes with different genomic backgrounds (Qi et al., 2013). Our previous study revealed a region subject to selection on chromosome 3, 8,194,851–11,520,071 (9930 v2), which includes *CsLH* (Liu et al., 2019). Given that *PHYB* genes have large genomic and functional diversity in plants (Karve et al., 2012; Li et al., 2015), we wondered whether the genomic diversity of *CsLH* in these groups conferred any adaptive advantages and was therefore selected for during cucumber breeding. To address this question, we used published genomic sequences from 115 cucumber accessions (Qi et al., 2013) to scan for a selection footprint. Notably, we found that the region surrounding *CsLH* (chr03:11088412–11,094,145) displayed reduced nucleotide diversity (π) in domesticated cucumber groups [East Asian (EA), Eurasian (EU), Xishuangbanna (XSBN), and Indian domestic (IND)] compared with the Indian wild genotypes (INW) (Fig. 1 j), suggesting that *CsLH* was selected during cucumber domestication. We then compared the SNP diversity in the *CsLH* genomic sequence among the EA, EU, XSBN, as well as the Indian populations (IN; IND + INW) (Fig. 1 k), revealing that IN, EA, and EU had 155, two, and one unique SNPs, respectively, while no unique SNPs were detected in XSBN. In addition, XSBN contained the least number of SNPs and the lowest nucleotide diversity (π *=* 0.858 × 10–3) (Fig. 1 k, l).

To investigate the genomic differentiation of *CsLH*, we calculated the pairwise difference in allele frequency (FST) between the domesticated groups and INW to identify differentially selected positions within each group. In EA, EU, and XSBN, a region subject to selection was identified in the first *CsLH* exon, specifically in the XSBN group, and it was evident that the entire N-terminus was under selection (Fig. 1 m). Given that the N-terminal domain of PHYB binds to the chromophore and shows photoreversible conformational changes, while mutations in the N-terminus result in altered gene function (Oka et al., 2008), it is possible that this selection in the N-terminus might have generated the distinct phenotypes between the different groups. In addition, a sequence analysis of the genomic region of *CsLH* in the 115 accessions identified 19 haplotypes (Supplementary Table 1). After removing the low-frequency (*n* ≤ 4) haplotypes, we identified three main haplotypes with six sequence polymorphisms in the *CsLH* ORF (Fig. 1 n). H1 was the most frequent haplotype detected among EA, EU, and IN, while XSBN only contained H2, suggesting that the XSBN-specific *CsLH* haplotype emerged during the long domestication process.

The above findings indicated that *CsLH* from the XSBN populations underwent a distinct selection separate to that of EA and EU. We therefore compared the hypocotyl lengths of *XSI48* (XSBN background), *9930* (EA background), and *LH* (EU background). XSI48 exhibited shorter hypocotyls than both 9930 and LH under light conditions (Fig. 1o–q), while all of them showed similar hypocotyl lengths in the dark (Fig. 1o’–q’), indicating that this phenotype is associated with light. In addition, quantitative reverse transcription (qRT)-PCR showed that XSI48 had a higher *CsLH* expression level than 9930 and LH in the dark (Fig. 1r). Given the different genetic backgrounds of XSI48, 9930, and LH, we cannot rule out that the short hypocotyl phenotype might be caused by other genes. To discriminate from other background effects, an introgression line named XSBN 3.2, based upon a 9930 background with the introgression of a segment from XSBN at chromosome 3, was created and used in this study (Fig. 1s). As shown in Fig. 1 T and 1 U, XSBN 3.2, exhibited a shorter hypocotyl length than 9930 under normal light conditions. This is consistent with the assertion that the *CsLH* allele from XSBN is responsible for the short hypocotyl.

## Discussion

We identified CsLH as a PHYB protein that regulates hypocotyl elongation in cucumber. *CsLH* underwent a distinct selection in different cucumber groups during domestication, revealing that a unique haplotype that conferred an adaptive advantage was selected during the breeding of XSBN. *CsLH* from XSBN could serve as a short hypocotyl regulatory gene with practical potential for creating short seedlings in cucumber breeding programs.

## Materials and methods

### Plant materials and growth conditions

The cucumber inbred lines Chinese Long 9930 (EA background); *LH* and its mutant *lh* (EU background); and XIS48 (XSBN background) were used in this study. The parental line Chinese Long 9930 was backcrossed three times to the F_1_ offspring of the Chinese Long 9930 × XIS48 cross, then selfed once to generate the introgression line XSBN3.2. The genotype of XSBN3.2 is shown in Supplementary Table 2.

The phenotypes of the XSBN3.2 seedlings were evaluated during the summer of 2020 (*n* = 12). The seeds were sterilized and soaked in water for 3 h, then transferred into Petri dishes lined with wet filter paper at 28 °C for 2 d. The seeds were then sown in plastic pots filled with autoclaved soil. The temperature was maintained at 26–30 °C (day) and 18–22 °C (night). The daytime light intensity was 195.1 μmol·m^− 2^·s^− 1^ ppfd.

### Hypocotyl measurement

The hypocotyl lengths of the seedlings were measured using a tape measure.

### Sequence alignment and phylogenetic analysis

The sequence alignment and phylogenetic analysis were performed on the sequences of the predicted PHY proteins from cucumber, Arabidopsis, and tomato. Multiple sequence alignments were performed using DNAMAN for Windows (Lynnon Corporation, San Ramon, California, USA). A phylogenetic tree was constructed using the maximum likelihood method.

### Genome resequencing, variant calling, and genotyping using KASPar

The genomic DNA of *LH*, *lh*, and XIS48 was extracted from leaves using a DNA isolation kit (Huayueyang biotech co., Ltd., Beijing, China). Genome resequencing was performed as described previously (Liu et al., 2019). In brief, at least 5 μg of DNA was prepared for library construction following the manufacturer’s instructions (Illumina, San Diego, California, USA). The raw data were filtered and aligned against the cucumber reference genome, Chinese Long 9930 (Li et al., 2019), to identify any differences. For the variant analysis, the assembled scaffolds were compared with the reference genome for the detection of SNPs and short insertion/deletion (InDels), which were then annotated. To test the role of *CsLH* at the population level, Chinese Long 9930 was crossed with the *lh* mutant to generate F_1_ generation plants, which were then self-fertilized to obtain an F_2_ population. A total of 2000 F_2_ individual plants were genotyped using a KASPar assay and the polymorphism of each marker was assessed using parental and F_1_ DNA. Primers for the SNP markers were designed using Primerpicker (LGC Genomics, Hoddesdon, UK), and their specificity was validated using a BLAST search on the cucumber reference genome (Li et al., 2019). The primer sequences used are listed in Supplementary Table 3.

### Gene cloning and expression analysis

The target sequence was cloned from genomic DNA and sequenced to confirm a 7-bp deletion within the *lh* mutant. Total RNA was extracted from the hypocotyls using an SV Total RNA Isolation System (Promega Corporation, Madison, Wisconsin, USA), and cDNA was synthesized using a MultiScribe™ reverse transcriptase kit (Thermo Fisher Scientific, Waltham, Massachusetts, USA) according to the manufacturer’s instructions. Primers for semi-quantitative RT- PCR and qRT-PCR were designed based on the 7-bp deletion. Cucumber *α-tubulin* (*TUA*) was used as an internal control. The primers used are listed in Supplementary Table 3.

### Cucumber transformation

To create the *plh: CsLH* construct, the promoter of *CsLH* was cloned from *lh* and inserted into the pEGAD vector between the *Stu*I and *Pac*I restriction sites. The predicted full-length *CsLH* cDNA was cloned and inserted into the same construct between the *Pac*I and *Sma*I restriction sites. The primers used are listed in Supplementary Table 3. The resulting construct was then introduced into *Agrobacterium tumefaciens* strain Agl-0 by electroporation and transformed into the cucumber *lh* mutant using a cotyledon transformation method with some modifications.

### Nucleotide diversity, selective footprint, and haplotype analysis

Resequencing data from 115 published cucumber accessions (37 EA, 29 EU, 19 XSBN, and 30 IN) were used for the analysis (Qi et al., 2013). Nucleotide diversity was estimated as an π value for each population using VCFtools, which was also used to estimate the fixation index (FST) between the four groups (XSBN, EA, EU, and IND) and their Indian wild counterparts (INW). After that, single nucleotides encoding variants (nonsynonymous and synonymous mutations) were selected to construct haplotypes using PHASE v2.1.1.

### Statistical analysis

All the treatments mentioned in this study involved at least three independent biological and technical replicates. The results were analyzed using analyses of variance (ANOVAs), and the significance of the differences between treatments was tested using Duncan’s test. All the analyses were carried out using Statistics Analysis System 15.1 (SAS Institute, Cary, North Carolina, USA) for Windows.

### Supplementary Information

**Additional file 1.**

**Additional file 2.**

**Additional file 3.**

## Data Availability

The authors declare that all the data and materials supporting the findings of this study are included in the main manuscript file or Supplementary Information or are available from the corresponding author upon request.
